# Detection and Characterization of *Leishmania* (*Leishmania*) and *Leishmania* (*Viannia*) by SYBR Green-Based Real-Time PCR and High Resolution Melt Analysis Targeting Kinetoplast Minicircle DNA

**DOI:** 10.1371/journal.pone.0088845

**Published:** 2014-02-13

**Authors:** Marcello Ceccarelli, Luca Galluzzi, Antonella Migliazzo, Mauro Magnani

**Affiliations:** 1 Department of Biomolecular Sciences, University of Urbino “Carlo Bo”, Fano (PU), Italy; 2 Istituto Zooprofilattico Sperimentale della Sicilia, Palermo (PA), Italy; 3 Department of Biomolecular Sciences, University of Urbino “Carlo Bo”, Urbino (PU), Italy; National Institute for Agriculture and Veterinary Research, IP (INIAV, I.P.), Portugal

## Abstract

Leishmaniasis is a neglected disease with a broad clinical spectrum which includes asymptomatic infection. A thorough diagnosis, able to distinguish and quantify *Leishmania* parasites in a clinical sample, constitutes a key step in choosing an appropriate therapy, making an accurate prognosis and performing epidemiological studies. Several molecular techniques have been shown to be effective in the diagnosis of leishmaniasis. In particular, a number of PCR methods have been developed on various target DNA sequences including kinetoplast minicircle constant regions. The first aim of this study was to develop a SYBR green-based qPCR assay for *Leishmania (Leishmania) infantum* detection and quantification, using kinetoplast minicircle constant region as target. To this end, two assays were compared: the first used previously published primer pairs (qPCR1), whereas the second used a nested primer pairs generating a shorter PCR product (qPCR2). The second aim of this study was to evaluate the possibility to discriminate among subgenera *Leishmania* (*Leishmania*) and *Leishmania* (*Viannia*) using the qPCR2 assay followed by melting or High Resolution Melt (HRM) analysis. Both assays used in this study showed good sensitivity and specificity, and a good correlation with standard IFAT methods in 62 canine clinical samples. However, the qPCR2 assay allowed to discriminate between *Leishmania* (*Leishmania)* and *Leishmania* (*Viannia)* subgenera through melting or HRM analysis. In addition to developing assays, we investigated the number and genetic variability of kinetoplast minicircles in the *Leishmania (L.) infantum* WHO international reference strain (MHOM/TN/80/IPT1), highlighting the presence of minicircle subclasses and sequence heterogeneity. Specifically, the kinetoplast minicircle number per cell was estimated to be 26,566±1,192, while the subclass of minicircles amplifiable by qPCR2 was estimated to be 1,263±115. This heterogeneity, also observed in canine clinical samples, must be taken into account in quantitative PCR-based applications; however, it might also be used to differentiate between *Leishmania* subgenera.

## Introduction

Leishmaniasis is a neglected disease of the Old and New Worlds with a broad clinical spectrum encompassing asymptomatic infection and three main clinical syndromes: visceral leishmaniasis (VL), cutaneous leishmaniasis (CL), and mucosal leishmaniasis (ML). Worldwide, at least 15 *Leishmania* species are pathogenic for *Homo sapiens*. They are primarily transmitted by phlebotomine sandflies, although infection may also occur sporadically through blood transfusion, contaminated needles, and organ transplantation [1]. *Leishmania donovani* complex (including *L. infantum* and *L. donovani*) belongs to the subgenus *Leishmania* (*Leishmania)* and it is the etiological agent of VL, while the species belonging to the subgenus *Leishmania* (*Viannia)* are the etiological agents of CL and ML. The leishmaniasis is still a public health problem in 98 countries, affecting both rural and urban areas. Worldwide there are an estimated 0.2–0.4 million new cases of VL and 0.7–1.2 million new cases of CL annually, while 12 million people are currently affected by the disease [2]. The VL mortality is second only to malaria among parasitic diseases [3].

In zoonosis caused by *L. (L.) infantum*, dogs are the main reservoir for the disease and canine visceral leishmaniasis (CVL) is considered to be a major problem in veterinary medicine [4], [5]. Cases of canine leishmaniasis have been reported in over 50% of the countries where human leishmaniasis is endemic, and the Mediterranean Basin is one of the most affected areas [6]. Moreover, many infected dogs are asymptomatic [7], [8].

In this context, efficient and reliable diagnostic approaches in veterinary medicine are very important in the treatment of symptomatic and asymptomatic dogs. Moreover, the definition of the subgenus/complex in humans may be useful in helping physicians to choose the appropriate therapeutic protocols and in gaining insights into the disease evolution [9].

To this end, many diagnostic systems have been developed. Among the serological methods, the indirect fluorescence antibody test (IFAT) is considered by the World Organization for Animal Health (OIE-Office International des Epizooties) as a reference serologic method [10]. Nevertheless, serological methods are not always reliable when dealing with prepatent periods, remission stages, and the appearance of non-specific cross-reactions or latent forms of the disease like in “cryptic leishmaniasis” [11]. Moreover, in endemic areas, a significant number of animals display antibody titres ranging from 1∶40 to 1∶80 (below the positivity threshold: titre ≥1∶160), referred to as uncertain titres or “borderline titres” [12].

Molecular techniques can lead to improvements in the diagnosis of leishmaniasis; in particular quantitative PCR (qPCR) could play an important role in *Leishmania* detection and the monitoring of therapy [13], [14] in humans and animals. Several PCR methods have been developed on various *Leishmania* target sequences. The conserved region of *Leishmania* kinetoplast DNA (kDNA) minicircles has been used as a specific target for conventional or quantitative PCR assays [15]–[17]. In fact, *Leishmania* belongs to the Kinetoplastida order, Trypanosomatidae family, in which all the members contain a kinetoplast situated at the base of the flagellum. The kinetoplast contains a concatenated network of circular DNA molecules [18], i.e. mitochondrial DNA, composed of minicircles and maxicircles. The minicircles, which encode for guide RNAs (gRNAs) required for editing the mRNA from maxicircles, have been reported to be present in about 10,000 copies per parasite [19], [20]. Structurally, the kDNA minicircle is organized into one to four conserved regions representing approximately 10% of the molecule and an equal number of variable regions [21].

In this study, we compared two SYBR green–based qPCR assays (named qPCR1 and qPCR2), targeting the kDNA minicircle constant region, for the detection and estimation of the *Leishmania* parasites in canine clinical samples. Then, we evaluated the possibility to discriminate among the subgenera *Leishmania* (*Leishmania*) and *Leishmania* (*Viannia*) using a high resolution melt (HRM) approach. Moreover, the number of kDNA minicircles and their genetic variability were also investigated in an attempt to characterize the *L. (L.) infantum* WHO reference strain and gain insight the minicircle heterogeneity in veterinary clinical samples.

## Materials and Methods

### Ethical Statement

Approval of the study was obtained on July 31^st^ 2012 from the Ethical Committee for Animal Experiments of the University of Urbino (CESA). The study’s title was “Diagnosi biomolecolare della leishmaniosi attraverso l’uso di campioni clinici non invasivi e loro utilizzo per il monitoraggio terapeutico” (Prot. CESA 2/2012).

### *Leishmania* DNA

A Chelex-purified DNA from promastigotes of *L. (L.) infantum* MHOM/TN/80/IPT1 (WHO international reference strain), used in Italy as the national reference strain, was obtained from the Institute of Experimental Preventive Veterinary Medicine (Istituto Zooprofilattico Sperimentale) (IZS) of Sicily, the National Italian Reference Centre for leishmaniasis located in Palermo, Italy. The equivalent concentration of reference sample was 10^8^ parasites/ml. DNA quantification was performed by fluorimetric analysis using the Qubit 2.0 Fluorometer (Invitrogen).

The DNA concentration was 23.5 ng/µl, and the content of DNA per cell was calculated to be 235 fg/parasite, in agreement with literature data [22], [23].This value confirmed the accuracy of parasite concentration in the DNA reference sample, and supported the accuracy of the subsequent determinations and quantifications.

Chelex-purified DNA from New World Leishmanias *L. (L.) amazonensis, L. (V.) guyanensis, L. (V.) panamensis, L. (V.) braziliensis* were also obtained from the same Institution. These strains were isolated from clinical samples in Argentina and typed at the species level at the Institute of Biomedicine and molecular immunology, CNR (Palermo, Italy). The DNA concentration of the New World *Leishmania* species was also analyzed, and the following results were obtained: *L. (L.) amazonensis* 0.98 ng/µl, *L. (V.) guyanensis* 2.31 ng/µl, *L. (V.) panamensis* 1.79 ng/µl and *L. (V.) braziliensis* 1.32 ng/µl.

### Canine Samples

Canine peripheral blood and conjunctival swabs samples were provided by the veterinary clinic “S. Teresa” (Fano, Italy) as part of samples used for routine clinical tests. Sixty-two animals were selected and grouped on the basis of IFAT test results and clinical signs reported by veterinary practitioners (i.e. lymphadenopathy, alopecia, skin ulceration, weight loss, onychogryposis, ocular lesions, epistaxis, lameness). IFAT was performed on serum samples, obtained from both symptomatic and asymptomatic dogs, with an in-house assay validated and provided by IZS of Sicily.

The buffy-coat samples (100 to 320 µl) were obtained after centrifugation of 1 ml peripheral blood at 1500 rpm for 10 minutes. The DNA was extracted from these samples using the DNeasy Blood & Tissue kit (Qiagen) following the manufacturer’s protocol with some slight modifications. In particular, the incubation time with proteinase K was prolonged to 2 h, and the elution was repeated twice with the same 200 µl elution buffer. Conjunctival swabs were collected from the right and left conjunctivas using sterile cotton swabs. The swabs were transferred into 1.5 ml sterile tubes, immersed in 200 µl lysis buffer (10 mM Tris-HCl pH 8.3, 50 mM KCl, 0.5% Nonidet P40, 0.5% Tween 20, 0.1 mg/ml proteinase K), and incubated 2 h at 56°C. After swabs elimination, the samples were incubated for 10 min at 95°C and centrifuged at 14,000 rpm for 10 min. Supernatants were used as template in PCR reactions.

### PCR Assays

**Figure 1:**
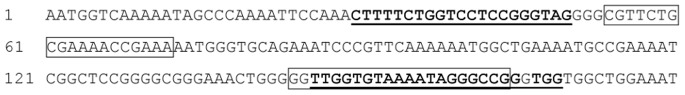
*L. (L.) infantum* kDNA minicircle DNA partial sequence (acc. n. Z35272) and primer localization. The underlined bold sequences represent the MaryF-MaryR primers; the boxed sequences represent MLF-MLR primers.

The primers used to amplify a 140 bp conserved region of the *Leishmania* kDNA minicircle were from Mary *et al.* [15] (defined in this paper as MaryF and MaryR). Two new primers (forward MLF: 5′-CGTTCTGCGAAAACCGAAA-3′; and reverse MLR: 5′-CGGCCCTATTTTACACCAACC-3′) were designed to target a 111 bp fragment of the same region of *L. (L.) infantum* kDNA minicircle (acc. n. Z35272) using the Primer Express software (Applied Biosystems). The positions of MLF-MLR primers as well as MaryF-MaryR primers along the kDNA minicircle sequence are depicted in Fig. 1.

Conventional PCR using both primer pairs was carried out in a 50 µl volume with 25–50 ng template DNA, containing 200 µM dNTPs, 2.5 mM MgCl_2_, 200 nM of each primer and 1U Hot-Rescue DNA Polymerase (Diateva). The amplification was performed in a GeneAmp^®^ PCR System 2700 (Applied Biosystems). The thermal cycling profile was as follows: 94°C for 7 min, followed by 35 cycles at 94°C for 30 s, 60°C for 20 s and 72°C for 20 s, with a final extension at 72°C for 5 min. Each sample was amplified in duplicate. Amplified fragments were analyzed by electrophoresis in a 1.8% agarose gel containing Gel Red (1∶10,000) (Sichim, Italy). The gels were visualized under UV light using a gel doc apparatus (Bio-Rad).

Two qPCR assays were named as qPCR1 and qPCR2 and were performed using MaryF-MaryR primers and MLF-MLR primers, respectively. Both qPCR were carried out in a 25 µl volume with 1 µl template DNA and 24 µl SYBR green PCR master mix (Diatheva srl, Italy) containing 1U Taq Polymerase and 200 nM of each primer. The PCR reactions were performed in a Rotor-Gene 6000 instrument (Corbett life science, Australia). The amplification profile was: 94°C for 10 min, followed by 40 cycles at 94°C for 30 s, 60°C for 20 s and 72°C for 20 s. At the end of each run, a melting curve analysis was performed from 55°C to 95°C to monitor primer dimers or non-specific product formation. The reactions were performed in duplicate or triplicate.

A standard curve was established using Chelex-purified *L. (L.) infantum* DNA; 1 µl of serial dilutions, ranging from 100 to 0.001 parasites, was introduced into reaction tubes. The standard curve concentration was expressed as parasite/µl (par/µl).

In order to evaluate the potential interference of host DNA as background in the qPCR analysis, we spiked the qPCR reactions, containing *L. (L.) infantum* DNA from 100 to 0.001 parasites, with 100 ng or 30 ng of human or canine DNA, respectively. The amount of canine DNA approximately reflected the median amount of DNA from clinical samples used as templates. All quantification analyses were performed with the Rotor-Gene 6000 software. Primer sequence specificity was confirmed *in silico* by BLAST searches in the subset database order Kinetoplastida. The assay specificities were also tested with DNA purified from *Trypanosoma cruzi*, obtained from the Institute of Biomedicine and Molecular Immunology, CNR (Palermo, Italy). To exclude false-negative results due to low DNA extraction efficiency or the presence of PCR inhibitors, a random subset (approximately 24%) of canine DNA samples which resulted qPCR-negative were tested for the quantitative amplification of the beta-2-microglobulin (B2M) gene using primers B2Mcanis_F (5′-GTCCCACAGATCCCCCAAAG-3′) and B2Mcanis_R (5′- CTGGTGGATGGAACCCTGAC-3′) with qPCR conditions as reported above.

### High Resolution Melt (HRM) Analysis

HRM curves acquisition was performed after PCR amplification on a Rotor-Gene 6000 instrument (Corbett life science, Australia). HRM range was set from 75°C to 92°C, with a slope of 0.1°C/s, and 2 s at each temperature. Each sample was run in duplicate or triplicate and gain was optimized before melt on all tubes. HRM curve analysis was performed with the derivative of the raw data, after smoothing, with the Rotor-Gene 6000 software.

To analyze intra-assay variation, the standard curve ranging from 100 to 0.01 *L. (L.) infantum* parasites, and spiked with 30 ng of canine DNA, was tested with 3 replicates within one run, and the coefficient of variation (CV) was calculated.

### Cloning and Sequencing

The PCR product amplified with primers MaryF-MaryR from the *L. (L.) infantum* MHOM/TN/80/IPT1 strain was cloned in the plasmid pCR®2.1 using the TA cloning Kit (Qiagen) and *E. coli* Top10 F competent cells. The recombinant plasmids were purified from five colonies (1, 2, 3, 4, 17) using QIAprep mini kit (Qiagen). The plasmid concentration was estimated using a gel doc apparatus (Biorad) by 1.8% agarose gel electrophoresis and λDNA/HinDIII marker (Thermo Scientific) as a reference. Plasmid copy number was calculated using the molar concentration and the molecular mass of the plasmid and the insert. The five clones were sequenced with M13 primers.

The PCR products obtained with MaryF-MaryR primers from canine clinical samples and New World Leishmanias were purified using the Minelute PCR purification kit (Qiagen) and directly sequenced using MaryF and MaryR primers.

All sequences were performed on a ABI PRISM 310 Genetic Analyzer (Applied Biosystems).

### kDNA Minicircle Quantification

The number of minicircles per parasite was determined in the *L. (L.) infantum* MHOM/TN/80/IPT1 strain by qPCR. Two standard curves were constructed with serial dilutions of plasmid 1 (ranging from 3.36×10^6^ to 3.36×10^3^ copies/PCR tube) or plasmid 3 (ranging from 3.59×10^6^ to 3.59×10^3^ copies/PCR tube) for amplification with primers MaryF-MaryR, or MLF-MLR, respectively.

### Statistical Analysis

Statistical analysis to evaluate differences among Tm values was performed using a Mann-Whitney test on GraphPad InStat (GraphPad Software, San Diego, CA).

## Results

### Specificity and Sensitivity of PCR Assays

**Figure 2:**
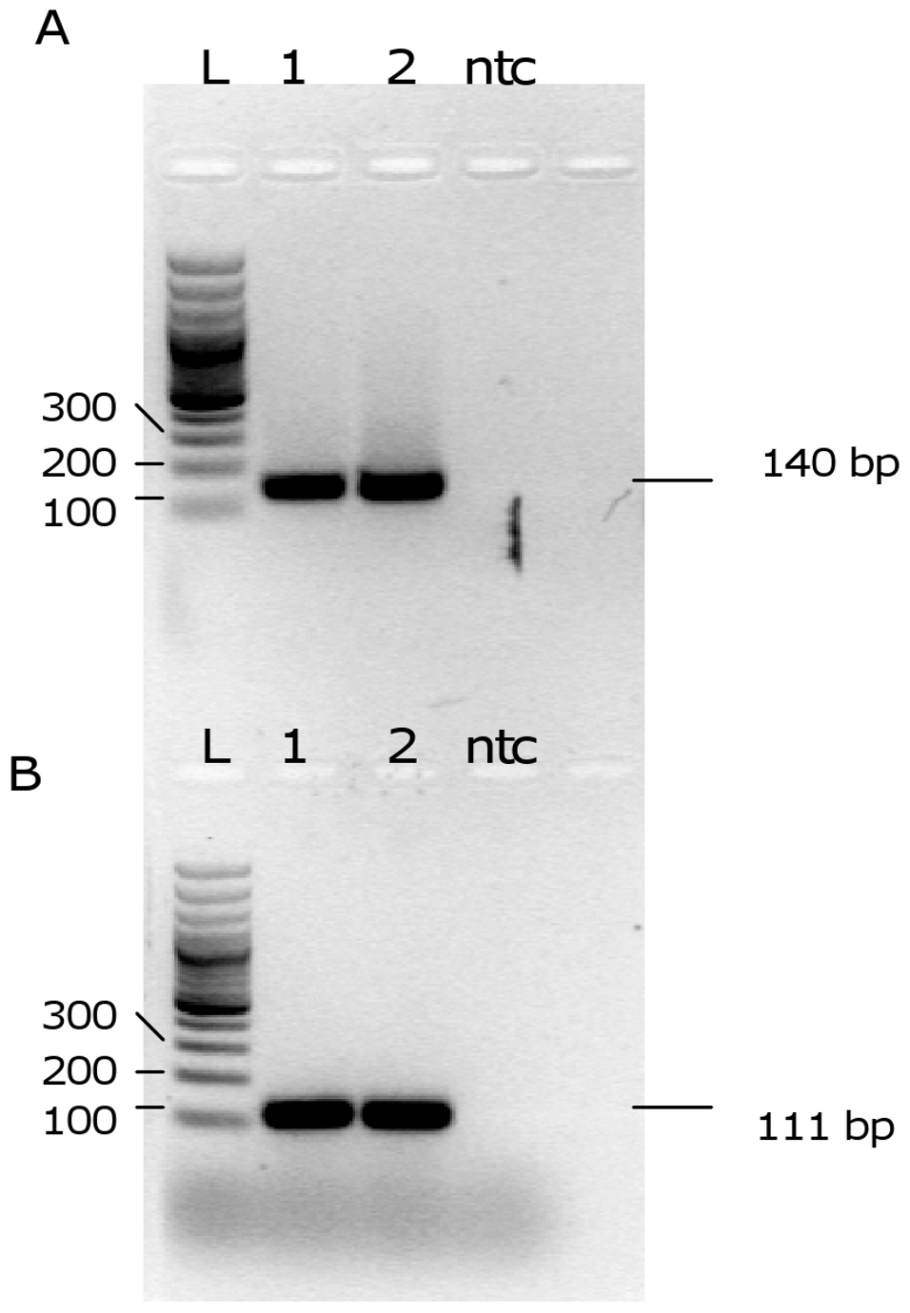
Electrophoretic analysis of PCR products. Agarose gel analysis of PCR products obtained with primers MaryF-MaryR (A) and MLF-MLR (B). 1,2: PCR products obtained with *L. (L.) infantum* MHOM/TN/80/IPT1 DNA as template; L: ladder; ntc: no template control.

Initially, to test the primers performance on DNA from *L. (L.) infantum* MHOM/TN/80/IPT1, 2.3 ng of template DNA were amplified by conventional PCR either with MaryF-MaryR primers and MLF-MLR primers. The electrophoretic analysis of the PCR mixtures showed the amplicons at the expected size (140 bp and 111 bp, respectively) and the absence of non-specific products or primer dimers (Fig. 2).

**Figure 3:**
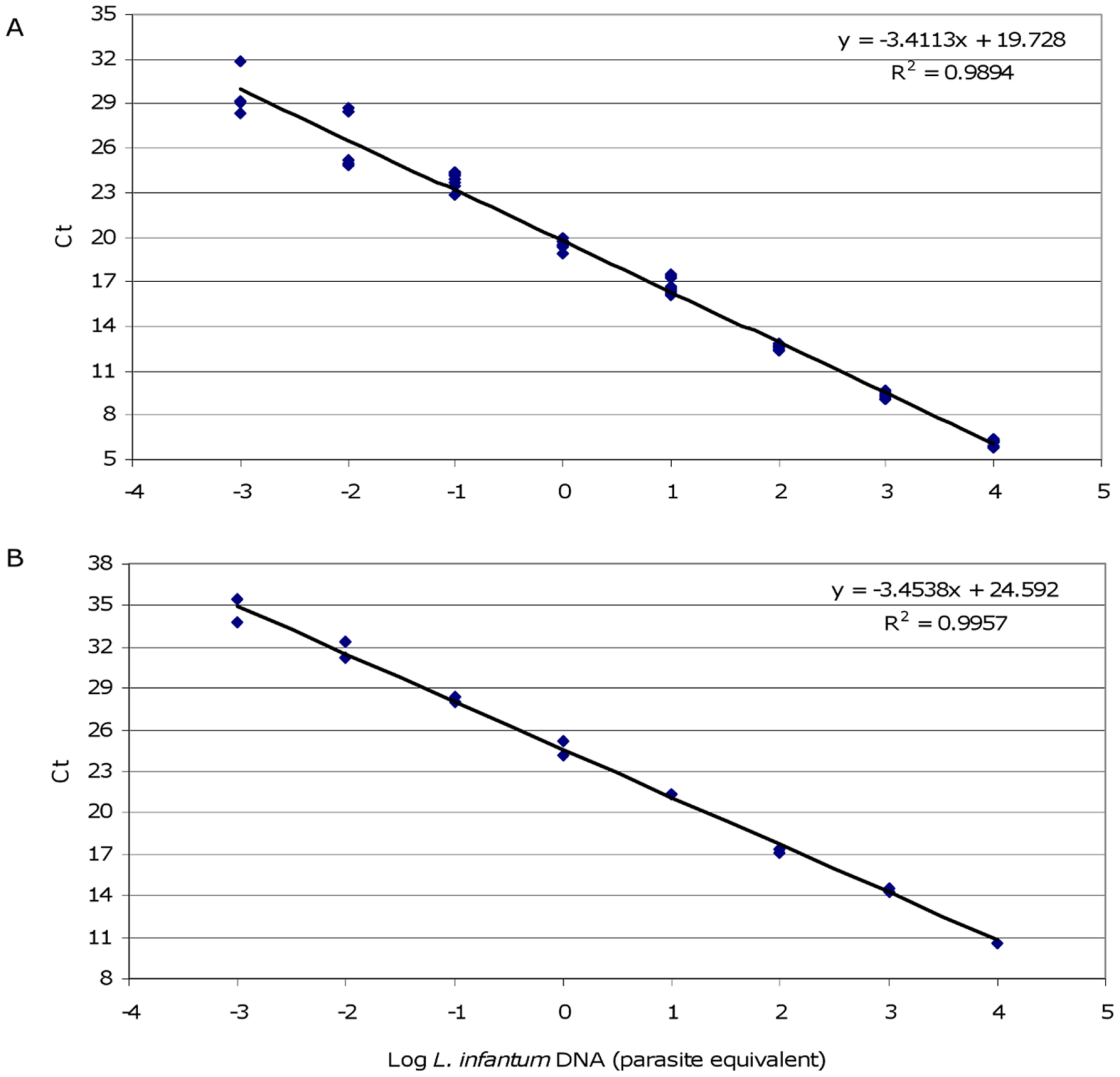
Calibration curves constructed with serial dilutions of *L. (L.) infantum* DNA. Standard curves obtained from serial dilutions of *L. (L.) infantum* MHOM/TN/80/IPT1 DNA with primers MaryF-MaryR and MLF-MLR are represented in panel A and B, respectively. The standard curves were obtained with serial dilutions ranging from 10,000 to 0.001 parasites equivalent/tube.

Subsequently, qPCR conditions with SYBR green were optimized with MaryF-MaryR and MLF-MLR primers: the results showed specific amplicons having melting temperatures of about 87°C and 85°C, respectively, without non-specific products or primer dimers (Fig. S1). Both qPCR assays showed a sensitivity of 1×10^−3^ parasites per PCR tube using calibration curves constructed with serial dilutions of *L. (L.) infantum* MHOM/TN/80/IPT1 DNA. Moreover, the PCR efficiencies were also similar (95% and 96%) (Fig. 3).

The specificity of both pairs of primers was tested with genomic human and canine DNA from healthy donors and with chelex-purified DNA from *L. (L.) infantum*, *L. (L.) amazonensis, L. (V.) guyanensis*, *L. (V.) panamensis*, *L. (V.) braziliensis,* and *Trypanosoma cruzi.* Human, canine or *T. cruzi* DNA did not show any amplification product (not shown). Both pairs of primers were able to amplify all the *Leishmania* species tested, including the New World species; however, in these species the Ct in the qPCRs were strongly delayed compared to *L. (L.) infantum* DNA, using comparable template DNA amounts (not shown). To exclude non-specific amplification, the same PCRs were performed with the annealing temperature at 65°C. The amplification products were also obtained under these more stringent conditions with both primer pairs (Fig. S2), indicating the existence of a subpopulation of kDNA minicircles matching primer sequences.

In order to exclude possible interference of background DNA derived from clinical specimens in qPCR assays, different amounts of *L. (L.) infantum* DNA (down to 1×10^−3^ parasites equivalent) were amplified in the presence of 100 ng of human DNA or 30 ng of canine DNA as background. Although the PCR efficiencies were affected by the presence of background DNA, 1×10^−3^ parasites were detected and the sensitivity of the assays remained unchanged (Fig. S3 and S4).

The qPCR2 sensitivities for *L. (L.) amazonensis, L. (V.) guyanensis*, *L. (V.) panamensis*, *L. (V.) braziliensis* were 1.0×10^−2^, 2.3×10^−3^, 2.0×10^−4^, 1.3×10^−3^ ng/PCR tube, respectively.

### kDNA Minicircle Quantification

**Figure 4:**
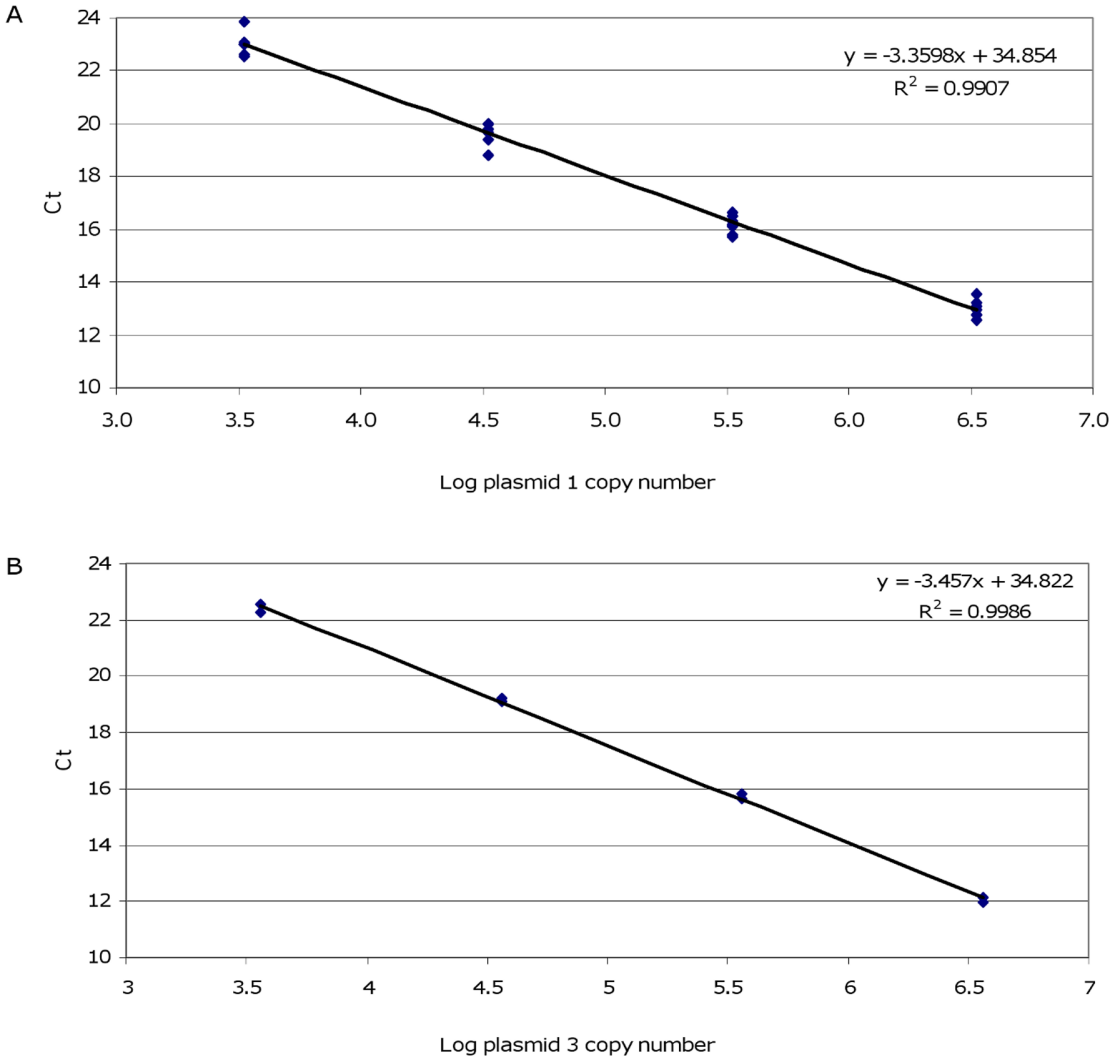
Calibration curves constructed with plasmid serial dilutions. Standard curves were obtained from serial dilutions of plasmid 1 (A) and plasmid 3 (B) with primers MaryF-MaryR and MLF-MLR, respectively. Each point derived from duplicates of 3 independent experiments.

kDNA minicircle quantification was first performed using MaryF-MaryR primers (qPCR1 assay). Serial dilutions of the PCR product cloned into a plasmid (plasmid 1), ranging from 3.36×10^6^ to 3.36×10^3^ copies, were used to construct the calibration curve. The kDNA minicircles were quantified in chelex-purified DNA equivalent to 10 and 1 par/µl. The quantification was performed in three independent experiments. The three standard curves were gathered, showing good correlation (Fig. 4A) and reproducible quantification results. We found 26,566±1,192 kDNA minicircles amplifiable by MaryF-MaryR primers in *L. (L.) infantum* MHOM/TN/80/IPT1.

The plasmid 1 was not amplifiable using MLF-MLR primers (data not shown). In fact, the cloned DNA sequence showed two mismatches with the MLF primer (see below). However, these primers successfully amplified a different cloned sequence (plasmid 3). Hence, serial dilutions of recombinant plasmid 3, ranging from 3.59×10^6^ to 3.59×10^3^ plasmid copies, were used in the construction of the calibration curve for kDNA minicircle quantification by MLF-MLR primers (fig. 4B). Dilutions of 100 and 10 par/µl were tested in duplicate obtaining an average of 1,263±115 kDNA minicircles per parasite amplifiable by MLF-MLR primers. Since the MLF-MLR primers are nested to MaryF-MaryR primers, we hypothesized that the 1,263±115 copies of this amplicon per cell, could represent a subclass of minicircles amplifiable by MaryF-MaryR primers.

### Melting Analysis

**Figure 5:**
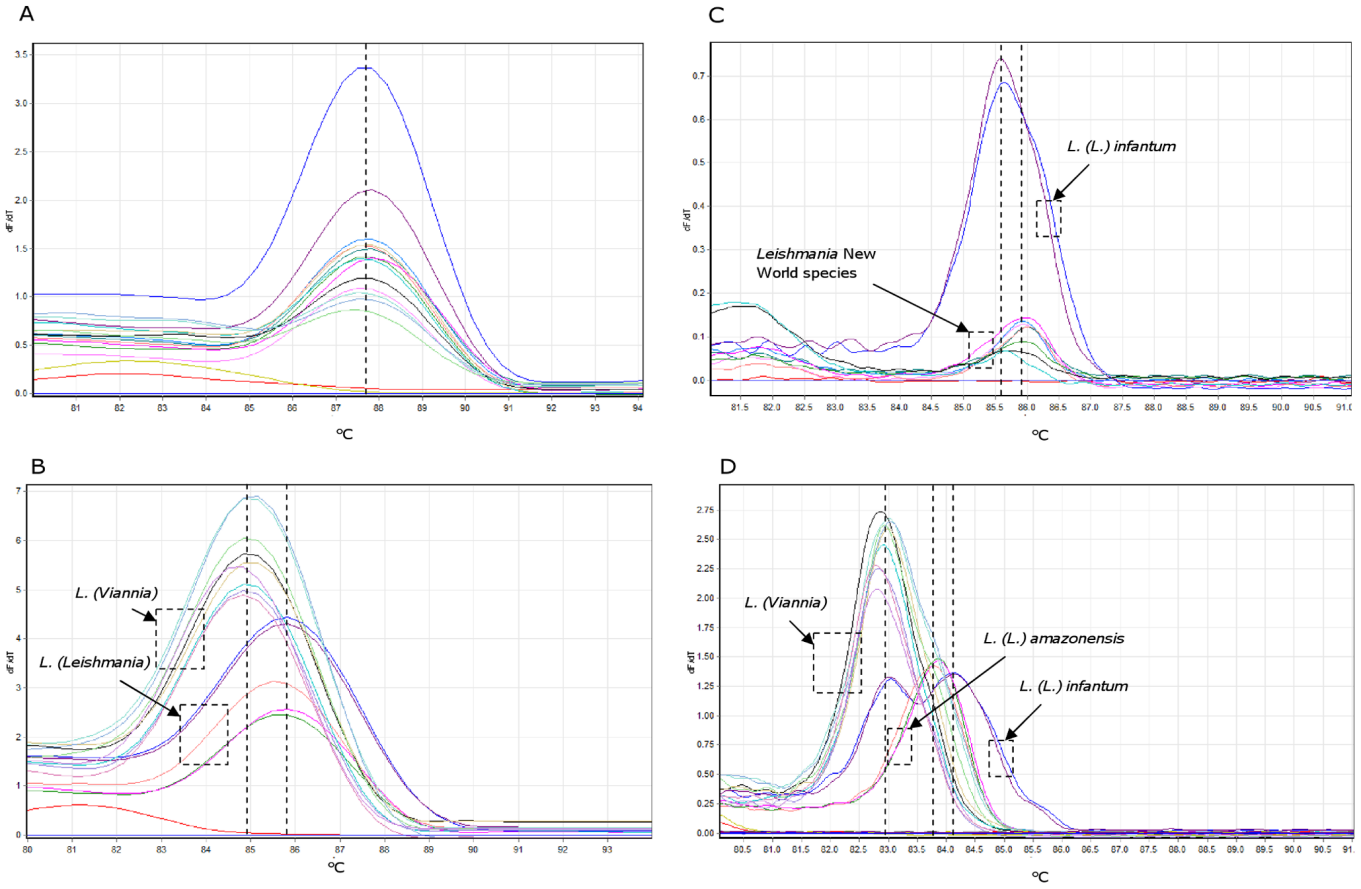
Melting analyses of PCR products. Representative melting profiles of amplicons obtained with primers MaryF-MaryR (A, C) and MLF-MLR (B, D). Panels A and B show results of standard melting analysis, while panels C and D show results of HRM analysis. The species tested were *L. (L.) infantum, L. (L.) amazonensis, L. (V.) guyanensis, L. (V.) panamensis*, *L. (V.) braziliensis*. Each species was tested in duplicate or triplicate. The melting curves which did not show a melting peak represent the no template controls.

Melting analysis of PCR products obtained with MaryF-MaryR primers (qPCR1) did not show significant Tm differences for the two subgenera *Leishmania (Leishmania)* (average Tm 87.62±0.18; n = 10) and *Leishmania* (*Viannia*) (average Tm 87.67±0.16; n = 9), while PCR products obtained with MLF-MLR primers (qPCR2) showed two significantly different Tm values for the subgenera *Leishmania* (*Leishmania)* (average Tm 85.74±0.25; n = 16) and *Leishmania* (*Viannia*) (average Tm 85.01±0.24; n = 26) (Mann-Whitney test p<0.0001) (Fig. 5AB). This observation was further strengthened by performing HRM analysis on the same samples (Fig. 5CD): the Tm analysis performed with MaryF-MaryR primers was still unable to efficiently discriminate among the different subgenera, while the assay with MLF-MLR primers showed significantly different HRM profiles for species belonging to subgenus *Leishmania* (*Viannia)*, for *L. (L.) amazonensis,* and for *L. (L.) infantum* MHOM/TN/80/IPT1 (Mann-Whitney test p<0.0001). Average Tm values obtained with HRM analysis from 32 replicates are shown in Table 1. These results suggest that it is possible to discriminate between *Leishmania* (*Viannia)* and *Leishmania* (*Leishmania)* subgenera by performing real-time PCR followed by melt or HRM analysis, corroborating similar results previously obtained by Pita-Pereira *et al*. [24], obtained with Brazilian strains. Moreover, using HRM analysis we were also able to discriminate between the reference strain *L. (L.) infantum* MHOM/TN/80/IPT1, showing a characteristic double peak, and *L. (L.) amazonensis,* showing a single peak (Fig. 5D) (Table 1). The HRM intra-assay analysis showed good reproducibility up to 0.1 parasite equivalent/reaction (average Ct ∼31) in samples containing 30 ng canine DNA as background (Table 2). Below this parasite concentration, the CV values were sensibly higher and the Tm of the peaks appeared slightly shifted.

**Table 1:** HRM analysis with MLF-MLR primers of different *Leishmania* species.

Species	subgenus	Tm peak 1 (°C)	Tm peak 2 (°C)
*L. infantum*	*Leishmania*	83.20±0.47	84.32±0.34
*L. amazonensis*	*Leishmania*		84.09±0.26
*L. guyanensis*	*Viannia*	83.21±0.27	
*L. panamensis*	*Viannia*	83.07±0.09	
*L. braziliensis*	*Viannia*	83.08±0.21	
*L. infantum* Plasmid 3	*Leishmania*		84.02±0.12
*L. infantum* Plasmid 17	*Leishmania*	83.34±0.30	

Tm values are ± SD.

**Table 2:** Intra-assay reproducibility of the qPCR2 HRM analysis.

Parasite equivalents/reaction	Average Ct	Average Tm peak 1 (°C) ± SD	%CV	Average Tm peak 2 (°C) ± SD	%CV
100	20.86	83.04±0.06	0.03	84.14±0.05	0.06
10	23.99	83.09±0.05	0.06	84.13±0.09	0.11
1	27.44	83.15±0.11	0.13	84.23±0.03	0.03
0.1	31.10	83.24±0.10	0.11	84.49±0.08	0.09
0.01	33.77	83.72±0.47	0.56	84.42±0.70	0.83

### Canine Clinical Sample Analysis

A total of 62 different canine blood samples from the Marches region (Central Italy), where *L. (L.) infantum* is present as a veterinary parasite, were analyzed with IFAT and qPCR assays (Table 3). These samples were divided into 4 groups: diagnosed Leishmaniasis (17 samples); asymptomatic Leishmaniasis (21 samples); suspected Leishmaniasis (14 samples); monitored after therapy (10 samples). The samples showing IFAT titres ≥1∶160 were defined positive. All samples from dogs diagnosed with Leishmaniasis also showed positive results in qPCR assays, with the exception of 5 samples (22, 23, 24, 25, 28). The qPCRs were subsequently repeated in conjunctival swabs from these dogs, showing positive results (data not shown). Samples from dogs monitored after therapy showed a positive IFAT titre but qPCR did not reveal any parasites, except for samples 26, 27, which showed a low parasite load. Moreover, two samples (6 and 7) from IFAT negative asymptomatic dogs resulted positive in both qPCR assays. Approximately 24% of the canine samples which resulted negative in qPCR assays were tested for the amplification of the B2M gene. The B2M Ct values ranged from 24.13 to 25.07 (average was 24.42±0.34), showing amplifiability and homogeneity of the DNA amount in all the tested samples.

**Table 3:** Results from canine clinical samples.

			qPCR1	qPCR2		
Clinical status	Sample ID	IFAT titre	Parasite load (par/ml blood)	Parasite load (par/ml blood)	Tm HRM peak 1	Tm HRM peak 2
Diagnosed Leishmaniasis	1	1∶80	0.35	0.27	–	84.73±0.07
	2	n.a.	0.10	0.14	83.62±0.14	84.54±0.23
	3	≥1∶160	1.72	12.24	–	83.87±0.05
	4	≥1∶160	8.00	2.40	–	83.95±0.04
	5	≥1∶160	20.58	1.27	83.82±0.23	84.80±0.10
	8	≥1∶160	1.40	5.00	83.95±0.25	84.68±0.25
	9	≥1∶160	3.18	26.91	84.07±0.12	84.80±0.04
	21	≥1∶160	0.52	19.88	–	84.23±0.07
	22	≥1∶160	neg	n.a.	n.a.	n.a.
	23	≥1∶160	neg	n.a.	n.a.	n.a.
	24	≥1∶160	neg	n.a.	n.a.	n.a.
	25	≥1∶160	neg	n.a.	n.a.	n.a.
	28	≥1∶160	neg	neg	–	–
	29	≥1∶160	0.88	4.94	–	84.21±0.13
	30	≥1∶160	1.66	19.20	84.22 *	85.40±0.04
	31	≥1∶160	0.25	1.41	84.68±0.14	85.37 *
	32	≥1∶160	0.56	4.68	84.1±0.08	84.83±0.08
Asymptomatic Leishmaniasis	6	neg	22.27	10.40	83.03±0.07	83.94±0.08
	7	neg	4.26	6.45	83.71±0.06	84.45±0.11
	10	neg	neg	neg	–	–
	11	neg	neg	neg	–	–
	12	neg	neg	neg	–	–
	13	neg	neg	n.a.	n.a.	n.a.
	33	neg	neg	n.a.	n.a.	n.a.
	49	neg	neg	n.a.	n.a.	n.a.
	50	neg	neg	n.a.	n.a.	n.a.
	51	neg	neg	n.a.	n.a.	n.a.
	52	neg	neg	n.a.	n.a.	n.a.
	53	neg	neg	n.a.	n.a.	n.a.
	54	neg	neg	n.a.	n.a.	n.a.
	55	neg	neg	n.a.	n.a.	n.a.
	56	neg	neg	n.a.	n.a.	n.a.
	57	neg	neg	n.a.	n.a.	n.a.
	58	neg	neg	n.a.	n.a.	n.a.
	59	neg	neg	n.a.	n.a.	n.a.
	60	neg	neg	n.a.	n.a.	n.a.
	61	neg	neg	n.a.	n.a.	n.a.
	62	neg	neg	n.a.	n.a.	n.a.
Suspected Leishmaniasis	14	1∶40	neg	n.a.	n.a.	n.a.
	15	1∶40	neg	n.a.	n.a.	n.a.
	16	1∶80	neg	n.a.	n.a.	n.a.
	17	1∶80	neg	n.a.	n.a.	n.a.
	34	1∶40	neg	n.a.	n.a.	n.a.
	35	1∶80	neg	n.a.	n.a.	n.a.
	36	1∶40	neg	n.a.	n.a.	n.a.
	37	1∶40	neg	n.a.	n.a.	n.a.
	38	1∶40	neg	n.a.	n.a.	n.a.
	39	1∶80	neg	neg	–	–
	40	1∶40	neg	n.a.	n.a.	n.a.
	41	1∶40	neg	n.a.	n.a.	n.a.
	42	1∶80	neg	neg	n.a.	n.a.
	43	1∶40	neg	n.a.	n.a.	n.a.
Monitored after therapy	18	≥1∶160	neg	neg	–	–
	19	≥1∶160	neg	neg	–	–
	20	≥1∶160	neg	neg	–	–
	26	≥1∶160	0.29	26.21	83.30±0.71	84.23±0.81
	27	≥1∶160	0.21	neg	–	–
	44	≥1∶160	neg	neg	–	–
	45	1∶80	neg	n.a.	n.a.	n.a.
	46	≥1∶160	neg	n.a.	n.a.	n.a.
	47	≥1∶160	neg	n.a.	n.a.	n.a.
	48	≥1∶160	neg	n.a.	n.a.	n.a.

*only one replicate showed the melting peak.

neg: negative.

n.a.: not available.

HRM analyses using MLF-MLR primers were also performed in 15 canine clinical samples, always using *L. (L.) infantum* MHOM/TN/80/IPT1 DNA as reference. Despite some variability, the results allowed us to confirm the presence of the subgenus *Leishmania* (*Leishmania)* in all the samples tested. However, the presence of an HRM profile comparable to *L. (L.) infantum* MHOM/TN/80/IPT1 (double peak) was confirmed in 10 of 15 samples (Table 3). Representative melting profiles of clinical samples are depicted in Fig. S5.

### Genetic Variability of kDNA Minicircles

**Figure 6:**
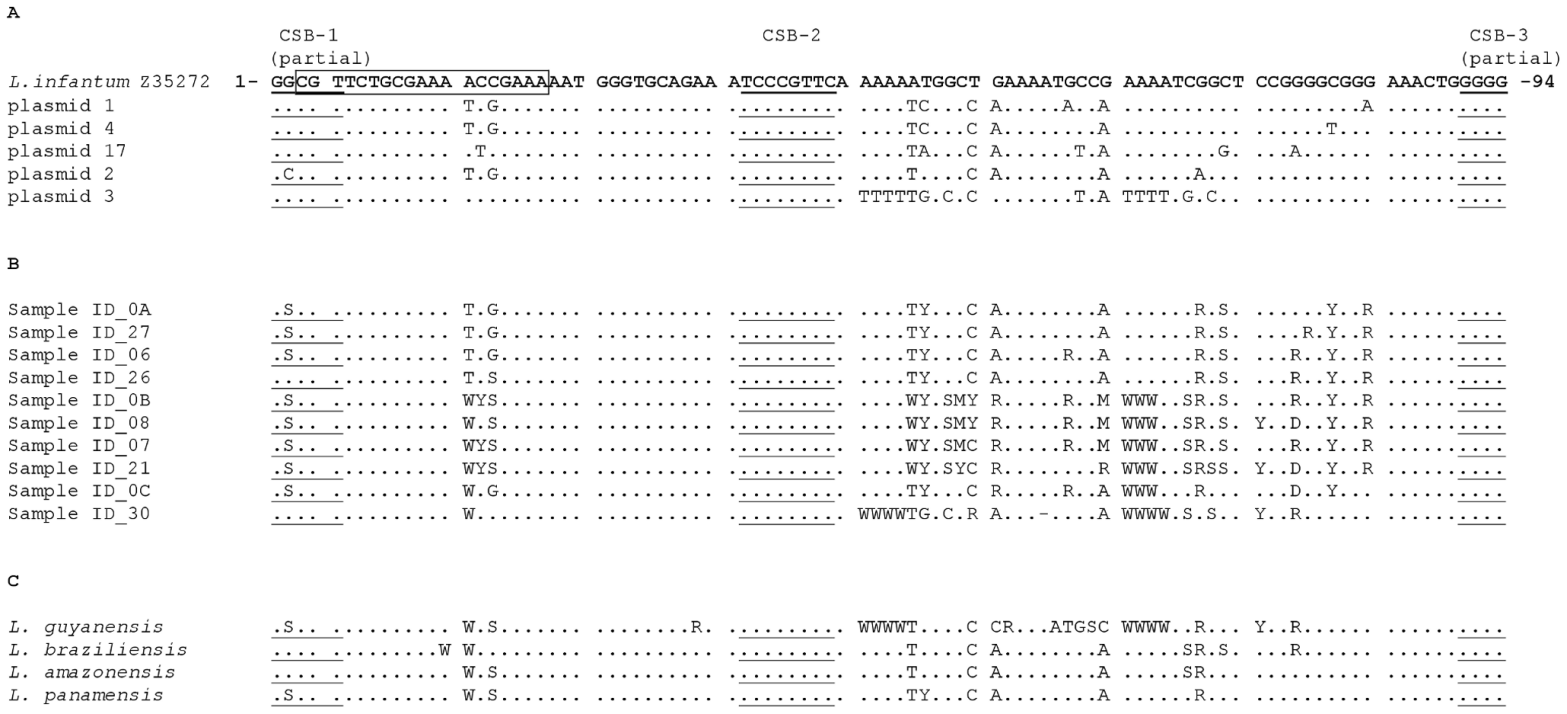
Sequence alignment of kDNA minicircle conserved region amplified with primers MaryF-MaryR. Panel A: alignment of five clones derived from *L. (L.) infantum* MHOM/TN/80/IPT1 strain. Panel B: alignment of PCR products from canine clinical samples. Panel C: alignment of PCR products obtained from New World *Leishmania* species. The boxed sequence represents the MLF primer. Underlined sequences represent CSBs box.

To investigate the genetic variability in the kDNA minicircle sequences amplified by qPCR1 assay, 5 cloned sequences obtained from *L. (L.) infantum* MHOM/TN/80/IPT1 were bidirectionally sequenced. Moreover, 10 amplicons obtained from canine clinical samples were directly sequenced. Comparing these nucleotide sequences by CLUSTALW2 [25], numerous polymorphic *loci* were highlighted both on plasmidic clones and on clinical samples (fig. 6AB). Interestingly, we also found a single base polymorphism G/C in the conserved sequence block 1 (CSB-1).

Regarding the New World species, the sequences of Mary’s amplicons were similar to *L. (L.) infantum* (fig. 6C), suggesting that Mary’s primers amplify a subclass of minicircles conserved among different subgenera or species.

## Discussion

A singular characteristic of the Kinetoplastida order is the mitochondrial DNA network organized in 20–50 maxicircles and 10,000–20,000 kDNA minicircles [21], [26]. With respect to the *Leishmania* genus, about 10,000 kDNA minicircles are estimated per parasite [19]. The conserved region of these minicircles has been used as a diagnostic PCR target since the 1990s [27]. Several genomic targets, other than the kDNA minicircle conserved region, have allowed species or complex differentiation in qPCR [28]–[30]. However, these assays may be less sensitive due to the lack of multiple copies of target sequence per cell.

The primers designed by Mary et al [15] have been widely used for *L. (L.) infantum* detection with the Taqman probe [31], [32]. We used these primers and a new primer pair (MLF-MLR) in a quantitative real-time PCR assay based on SYBR green chemistry. Good sensitivity, specificity and efficiency were obtained with both pairs of primers, although some inhibition was noted using an elevated amount (100 ng) of human DNA as background. Nevertheless, this inhibition did not affect the sensitivity of both assays.

These assays were used to make diagnoses in several canine blood samples, generally confirming the qualitative results obtained with IFAT. The discrepancies observed between IFAT and qPCR in samples from dogs diagnosed with Leishmaniasis (22, 23, 24, 25, 28) may be due to the low parasite content in blood compared to that which is found in bone marrow, lymphnodes or ocular conjunctiva [33]. In fact, a subsequent qPCR in conjunctival swabs from those dogs yielded positive results. On the contrary, the case of two samples that were qPCR positive and IFAT negative from asymptomatic dogs highlights the sensitivity of molecular methods, making this approach also useful for blood donor screening.

The discrepancies in quantification results between qPCR1 and qPCR2 may be explained by kDNA minicircle variability, in terms of number and sequence heterogeneity, in clinical samples. In fact, quantifications were performed using a standard curve obtained with DNA from the *L. (L) infantum* MHOM/TN/80/IPT1 strain.

Parasite quantification could be useful in follow-up, disease relapse or therapy monitoring. Parasite quantification by qPCR can be performed using standard curves obtained either with parasite serial dilutions or with dilutions of a cloned target sequence [34]. In this last case it is very important to know the amount of the PCR target per cell [35]. We attempted to quantify the amount of kDNA minicircles in *L. (L.) infantum* MHOM/TN/80/IPT1 WHO international reference strain with MaryF-MaryR primers and MLF-MLR primers using a cloned sequence as reference standard, resulting in about 26,000 and 1,200 copies per cell, respectively. The value obtained with MaryF-MaryR primers is greater than the value usually reported in literature [19], [34]. This may be due to the variability in kDNA minicircle number observed among different strains [15]. On the other hand, the value obtained with MLF-MLR primers represents a subclass of minicircles matching the primer sequences, as demonstrated by sequencing data. In fact, the heterogeneity of the kDNA minicircle conserved region was shown by sequencing five cloned sequences amplified with Mary’s primers (clones n. 1, 2, 3, 4, 17). The sequences were aligned, revealing several variations. Only clone n. 3 showed a perfect match with MLF-MLR primers, indicating that the kDNA minicircle sequence amplified by these primers was a subclass of the total kDNA minicircle population. This variability could make absolute quantification of *Leishmania* parasites in clinical samples difficult to achieve; however, the qPCR assays could still be useful for monitoring the relative changes of parasite loads during disease and/or therapy in clinical specimens from single patients.

The GC content in the Mary’s amplicons varied between 53.2% (clone n. 3) and 48.9% (clone n. 17), accounting for the two melting peaks observed in HRM analysis. Direct sequencing of qPCR1 amplicons from clinical samples confirmed the heterogeneity of these samples (Fig. 6B). It is worth noting the presence of a yet-to-be-described polymorphic site (G/C) in the conserved block 1 (CSB-1), which was confirmed with direct sequencing of PCR products from clinical samples. Due to the observed variability, SYBR green-based assays seem to be preferable to probe-based assays, unless the probe is designed on the 27 conserved nucleotides encompassing the CSB-2 region (Fig. 6) or it overlaps one of the primers.

These PCR assays were also used to test New World *Leishmania* species: *L. (L.) amazonensis, L. (V.) guyanensis*, *L. (V.) panamensis* and *L. (V.) braziliensis*. As represented in Fig. S2, primers MaryF-MaryR and MLF-MLR amplified all these New World species. However, a BLAST search highlighted the lack of sequence homology for MaryF-MaryR primers in *L. (V.) guyanensis*, *L. (V.) panamensis* and *L. (V.) braziliensis* kDNA minicircle sequences, and 4–7 mismatches in *L. (L.) amazonensis* kDNA minicircle sequences. In addition, MLF-MLR primers showed from 1 to 6 mismatches with these species’ sequences in Genbank database. Nevertheless, the kDNA minicircle sequences belonging to these species were also amplifiable under very stringent conditions (annealing temperature up to 65°C), indicating the presence of a subpopulation of minicircles with sequences matching the primers used. The fact that Mary’s primers can amplify New World species has recently been confirmed [36]. Taken together, these data suggest that Mary’s primers amplify a subclass of minicircles conserved among different subgenera or species. However, this does not explain the differences in Tm observed with MLF-MLR primers since the target of these primers belongs to a different subclass of minicircles.

When New World species DNA was used as a template, the qPCR2 assay resulted in lower Ct values than those found with the qPCR1 assay. Hence, it could be hypothesized that these New World species have more kDNA minicircles amplifiable (perfectly matching) by MLF-MLR primers respect to MaryF-MaryR primers.

The early characterization of the infecting parasite is important for appropriate treatment and evolution of the disease [9]. The ability to discriminate between the subgenera *Leishmania* (*Leishmania)* and *Leishmania* (*Viannia)* using real-time PCR and melt analysis targeting kinetoplast DNA has already been demonstrated in Brazilian *Leishmania* strains [24]. Here we confirmed that it is possible to discriminate between *Leishmania* (*Leishmania)* and *Leishmania* (*Viannia)* subgenera using MLF-MLR primers. The assays were performed on the *L. (L.) infantum* WHO reference strain and *L. (L.) amazonensis, L. (V.) guyanensis, L. (V.) panamensis, L. (V.) braziliensis* isolates (Fig. 5B).

Saturating fluorescent dyes such as LC Green, SYTO9 or Eva Green were generally considered necessary for HRM analysis [37]. However, also SYBR Green has proven to be a very successful dye for HRM analysis using the Rotor-Gene 6000 [38], [39], probably due to the technical features of this instrument (i.e. high thermal precision, short optical path, multiple readings for each thermal point). Therefore, performing HRM analysis with SYBR green, we were able to further confirm the Tm differences and to discriminate, in the subgenus *Leishmania* (*Leishmania)*, between *L. (L.) infantum* WHO reference strain and *L. (L.) amazonensis* (Fig. 5D). These results were also confirmed in 15 canine clinical samples from Central Italy: although clinical samples showed a greater variability in Tm, it was always possible to confirm the presence of *Leishmania* (*Leishmania)* DNA or to exclude the presence of *Leishmania (Viannia)* DNA. Moreover, in 10 of 15 samples, the HRM profile typical of the *L. (L.) infantum* WHO reference strain was also confirmed. The fact that five clinical samples did not show the first peak may be due to kDNA minicircle variability in the parasite. Samples that amplify late (Ct>30) or fail to reach a plateau in the PCR phase can result in inconclusive or low-resolution HRM data [40]. In our experience, reproducible Tm profiles were obtained when amplicons Ct values were approximately from 20 to 30. Out of this range, it should be difficult to compare Tm profiles (Table 2) and this could be a limit of the method when clinical samples with low parasite load are analyzed.

The fact that clinical sample DNA was in low-salt buffer and DNA from *L. (L.) infantum* MHOM/TN/80/IPT1 (used as positive control in all qPCR and HRM analyses) was Chelex-purified did not affect the analyses since chelex-purified DNA was diluted at least 1∶10,000 prior to be used as template and proved not to induce PCR inhibition.

In conclusion, the new SYBR green-based assay developed using MLF-MLR primers was shown to reliably detect *L. (L.) infantum* in canine clinical samples. Moreover, the kDNA minicircle constant region in the *L. (L.) infantum* WHO international reference strain was quantified and several polymorphic sites were highlighted. The qPCR2 followed by HRM analysis has shown to be able to discriminate between subgenera *Leishmania* (*Leishmania)* and *Leishmania* (*Viannia)*, confirming the results previously obtained in Brazilian strains, and to differentiate the *L. (L) infantum* WHO reference strain from *L. (L.) amazonensis*.

## Supporting Information

Figure S1 **Melting analysis of PCR products.** Melting temperature analysis of amplicons generated with primers MaryF-MaryR (A) and MLF-MLR (B) are shown. The Tm were 87.0°C and 85.3°C, respectively. Moreover, no dimers or non-specific products were detected.
(PPT)

Figure S2 **Conventional PCR under stringent conditions.** The PCR was conducted under stringent conditions (annealing temperature 65°C) with primers MaryF-MaryR (A) and MLF-MLR (B). 1: *L. (L.) infantum* (2.3×10^−4^ ng DNA/tube) (positive control); 2: *L. (L.) amazonensis* (1 ng DNA/tube); 3: *L. (V.) guyanensis* (2.3 ng DNA/tube); 4: *L. (V.) panamensis* (1.8 ng DNA/tube); 5: *L. (V.) braziliensis* (1.3 ng DNA/tube). All samples were tested in duplicate. A 100 bp-DNA ladder and a marker 9 (Fermentas) were used as reference in panel A and B, respectively.
(PPT)

Figure S3 **Standard curves with human DNA as background.** Standard curves were obtained from serial dilutions of *L. (L.) infantum* MHOM/TN/80/IPT1 DNA with primers MaryF-MaryR (A) and MLF-MLR (B) in the presence of 100 ng of human DNA per PCR tube. *L. (L.) infantum* DNA scalar dilutions were equivalent to 100, 0.1, 0.01 and 0.001 parasites/tube.
(PPT)

Figure S4 **Standard curves with canine DNA as background.** Standard curves were obtained from serial dilutions of *L. (L.) infantum* MHOM/TN/80/IPT1 DNA with primers MaryF-MaryR (A) and MLF-MLR (B) in the presence of 30 ng of canine DNA per PCR tube. *L. (L.) infantum* DNA scalar dilutions were equivalent to 100, 10, 1, 0.1, 0.01 and 0.001 parasites/tube.
(PPT)

Figure S5 **Representative HRM profiles of amplicons obtained with MLF-MLR primers (qPCR2) in clinical samples.** All samples are shown in duplicates. The melting profile of *L. (L.) infantum* MHOM/TN/80/IPT1 was always included as reference. The samples 3 and 4 show a single peak, corresponding to peak 2 of *L. (L.) infantum* (A). The samples 2, 6, 7, 8 show two peaks corresponding to peak 1 and 2 of *L. (L.) infantum* (B, C, D). Tm values are indicated in Table 3.
(PPT)
