# Supplementary figures and images for: Detection and Characterization of Leishmania (Leishmania) and Leishmania (Viannia) by SYBR Green-Based Real-Time PCR and High Resolution Melt Analysis Targeting Kinetoplast Minicircle DNA
Source: PLoS One. 2014 Feb 13;9(2):e88845. doi: 10.1371/journal.pone.0088845 (PMC3923818; doi:10.1371/journal.pone.0088845)

## Slide 1
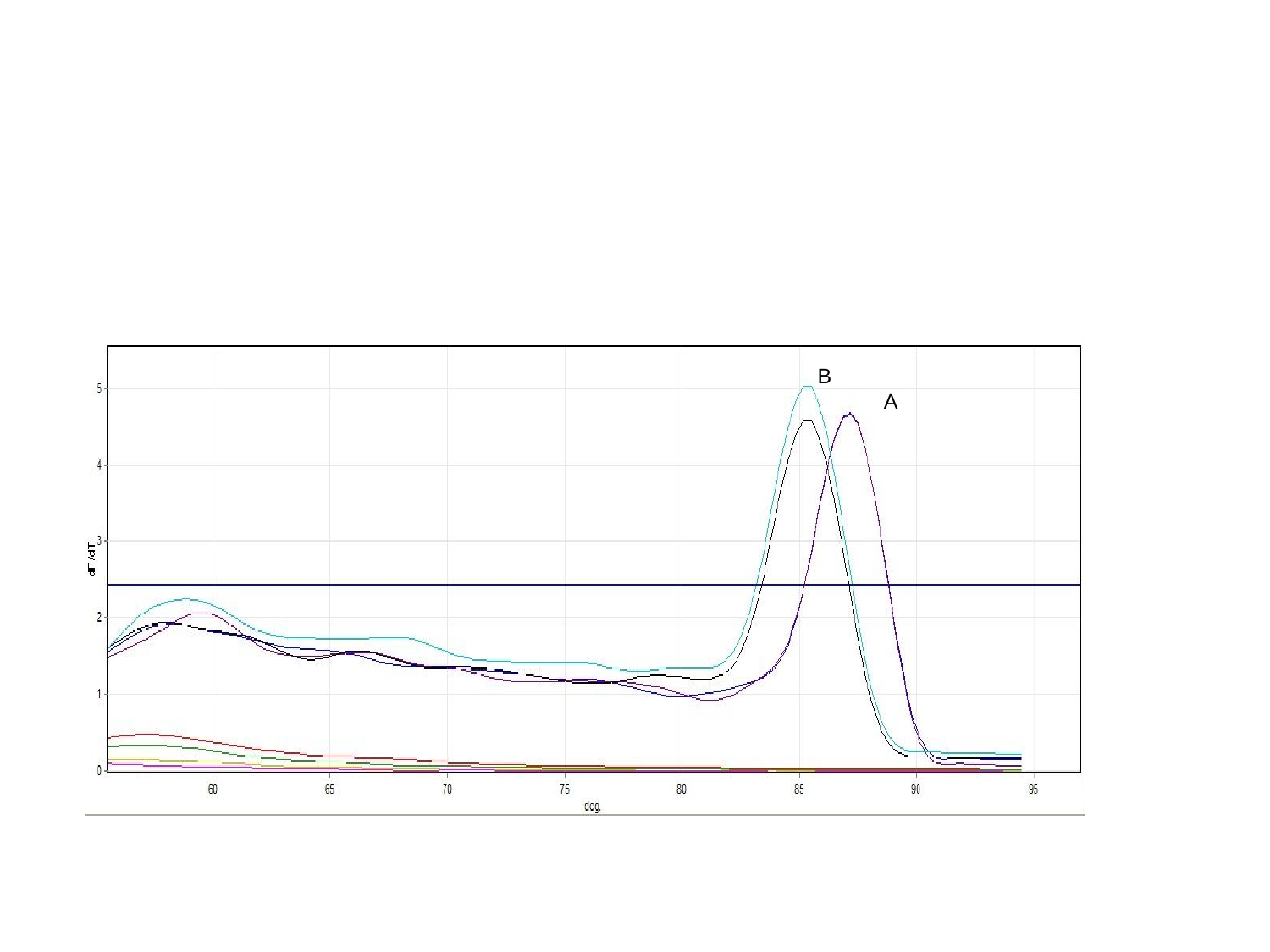

B
A

Supplement: Figure S1 — Melting analysis of PCR products. Melting temperature analysis of amplicons generated with primers MaryF-MaryR (A) and MLF-MLR (B) are shown. The Tm were 87.0°C and 85.3°C, respectively. Moreover, no dimers or non-specific products were detected. (PPT) [file pone.0088845.s001.ppt]

## Slide 1
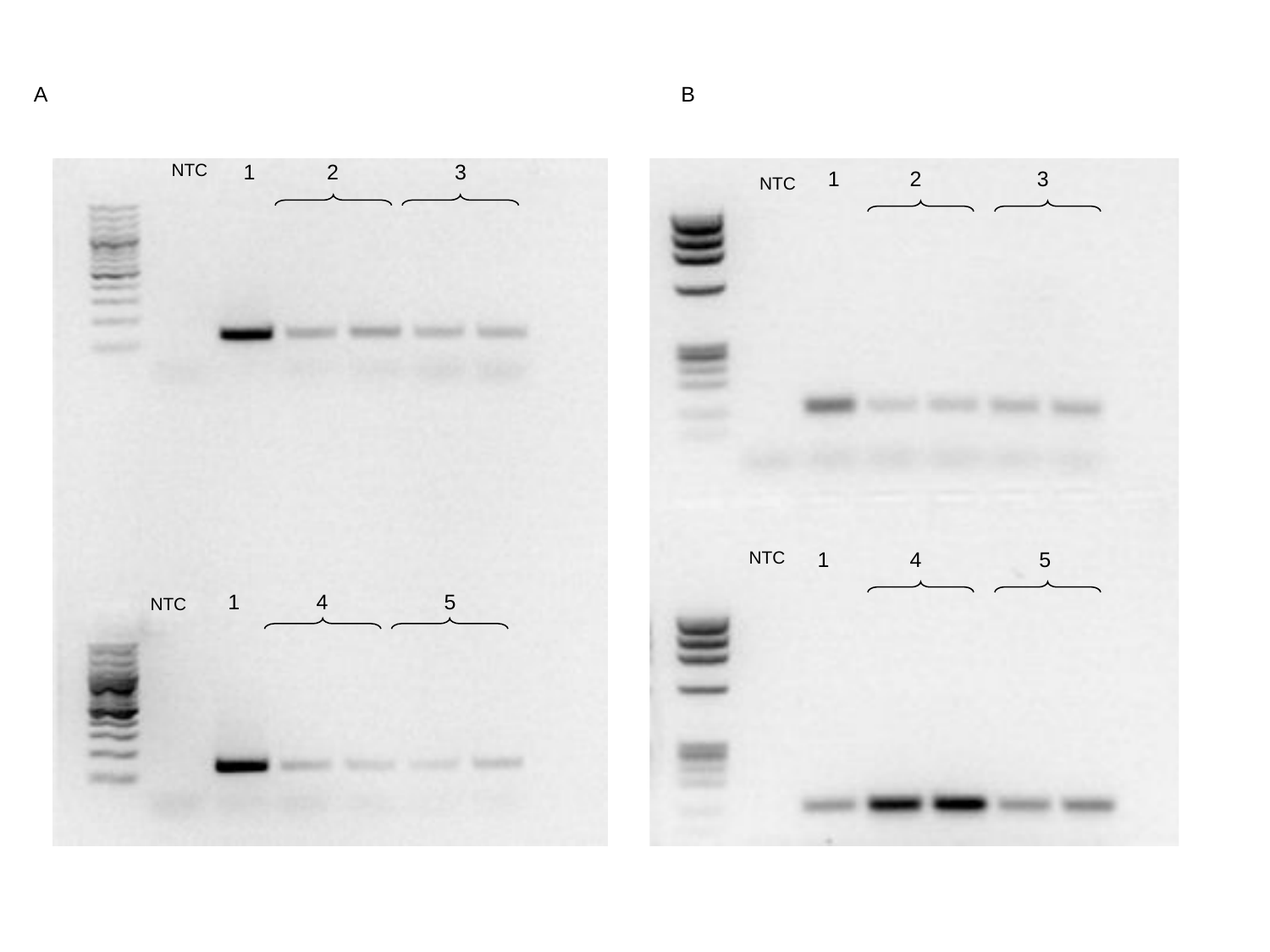

A
B
NTC
1
2
3
1
2
3
NTC
NTC
1
4
5
1
4
5
NTC

Supplement: Figure S2 — Conventional PCR under stringent conditions. The PCR was conducted under stringent conditions (annealing temperature 65°C) with primers MaryF-MaryR (A) and MLF-MLR (B). 1: L. (L.) infantum (2.3×10^−4^ ng DNA/tube) (positive control); 2: L. (L.) amazonensis (1 ng DNA/tube); 3: L. (V.) guyanensis (2.3 ng DNA/tube); 4: L. (V.) panamensis (1.8 ng DNA/tube); 5: L. (V.) braziliensis (1.3 ng DNA/tube). All samples were tested in duplicate. A 100 bp-DNA ladder and a marker 9 (Fermentas) were used as reference in panel A and B, respectively. (PPT) [file pone.0088845.s002.ppt]

## Slide 1
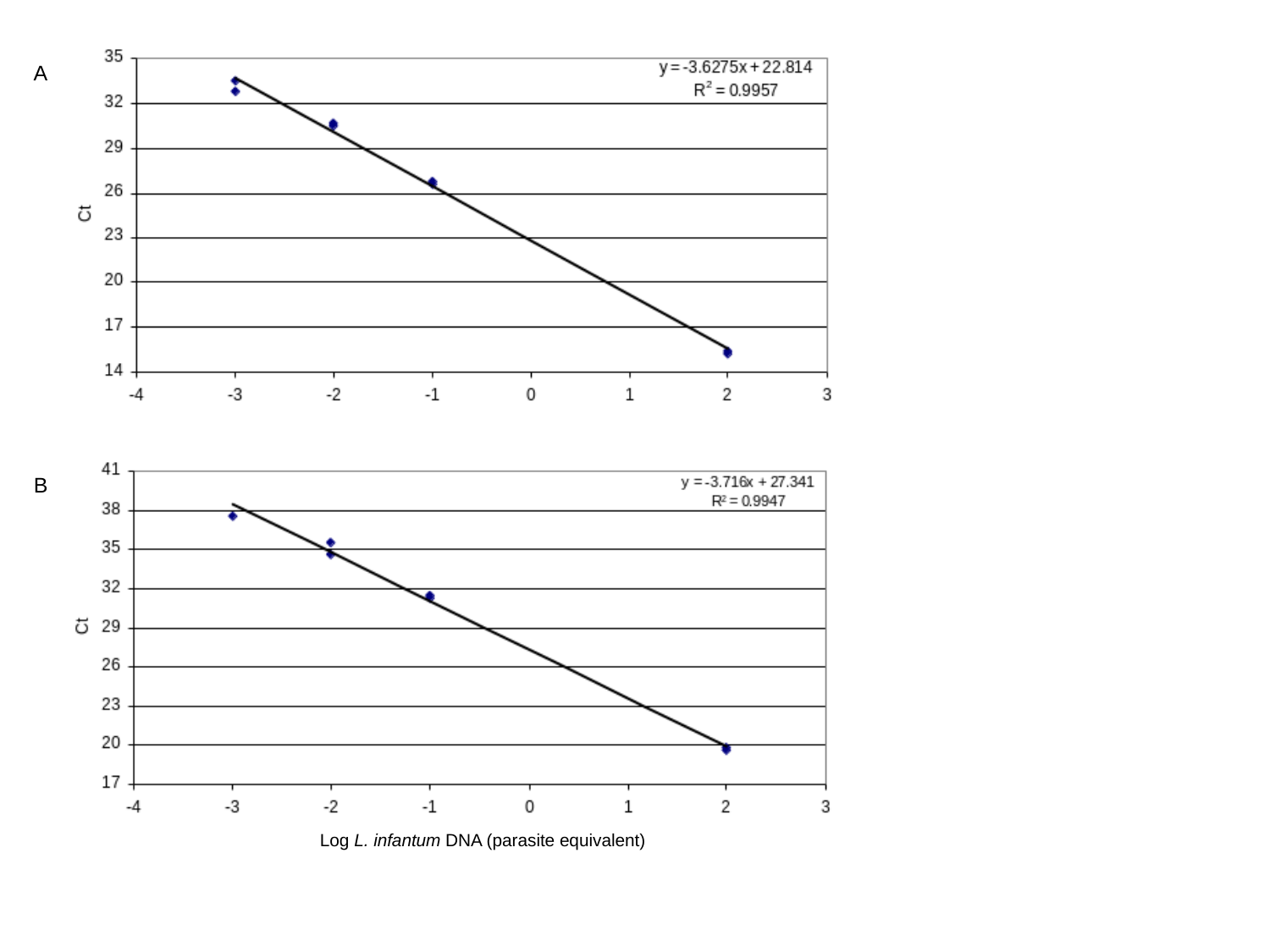

A
B
Log L. infantum DNA (parasite equivalent)

Supplement: Figure S3 — Standard curves with human DNA as background. Standard curves were obtained from serial dilutions of L. (L.) infantum MHOM/TN/80/IPT1 DNA with primers MaryF-MaryR (A) and MLF-MLR (B) in the presence of 100 ng of human DNA per PCR tube. L. (L.) infantum DNA scalar dilutions were equivalent to 100, 0.1, 0.01 and 0.001 parasites/tube. (PPT) [file pone.0088845.s003.ppt]

## Slide 1
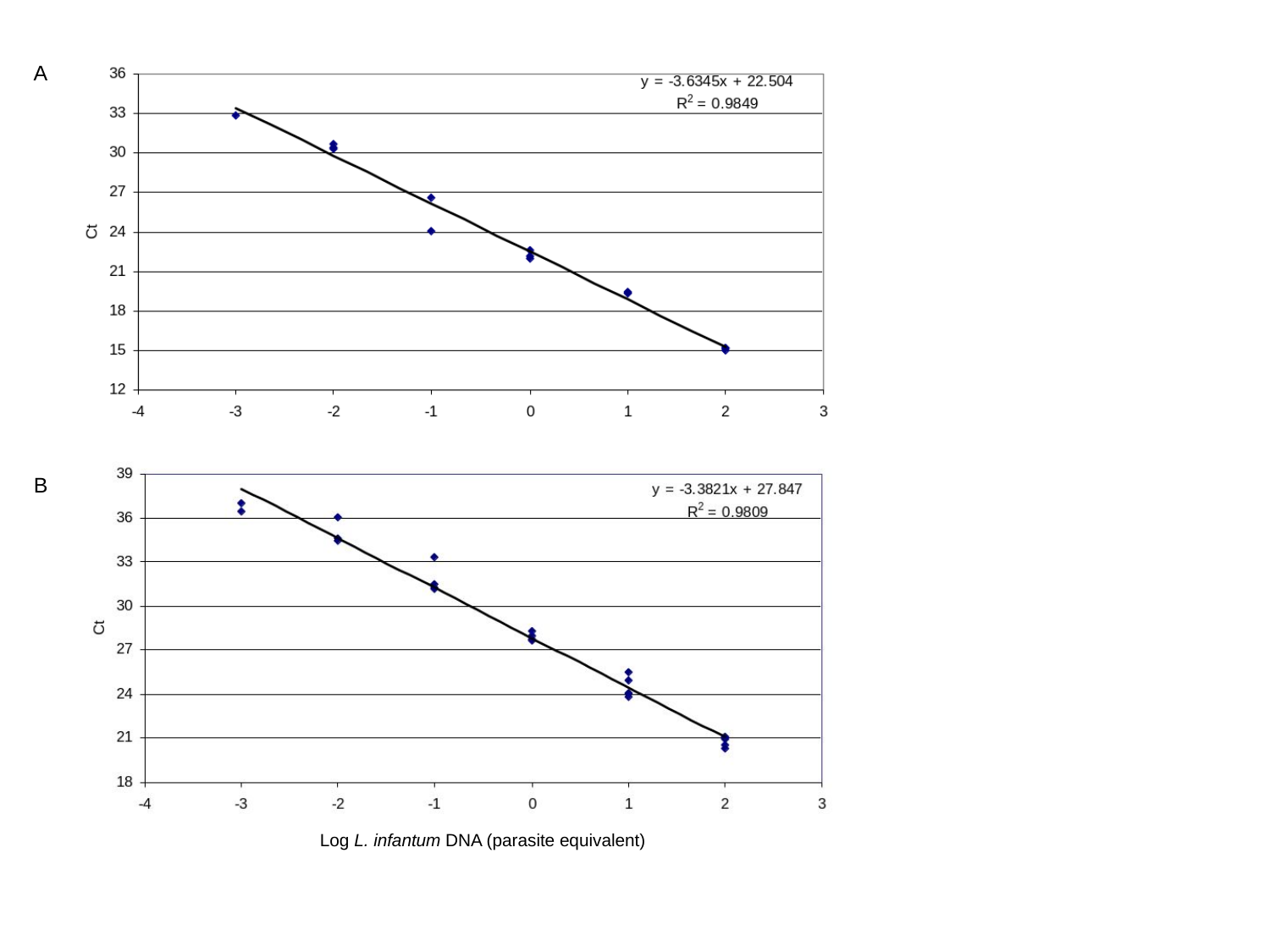

A
B
Log L. infantum DNA (parasite equivalent)

Supplement: Figure S4 — Standard curves with canine DNA as background. Standard curves were obtained from serial dilutions of L. (L.) infantum MHOM/TN/80/IPT1 DNA with primers MaryF-MaryR (A) and MLF-MLR (B) in the presence of 30 ng of canine DNA per PCR tube. L. (L.) infantum DNA scalar dilutions were equivalent to 100, 10, 1, 0.1, 0.01 and 0.001 parasites/tube. (PPT) [file pone.0088845.s004.ppt]
