# Supplementary material for: Detection and Characterization of Leishmania (Leishmania) and Leishmania (Viannia) by SYBR Green-Based Real-Time PCR and High Resolution Melt Analysis Targeting Kinetoplast Minicircle DNA
Source: PLoS One. 2014 Feb 13;9(2):e88845. doi: 10.1371/journal.pone.0088845 (PMC3923818; doi:10.1371/journal.pone.0088845)
Supplement: Figure S5 — Representative HRM profiles of amplicons obtained with MLF-MLR primers (qPCR2) in clinical samples. All samples are shown in duplicates. The melting profile of L. (L.) infantum MHOM/TN/80/IPT1 was always included as reference. The samples 3 and 4 show a single peak, corresponding to peak 2 of L. (L.) infantum (A). The samples 2, 6, 7, 8 show two peaks corresponding to peak 1 and 2 of L. (L.) infantum (B, C, D). Tm values are indicated in Table 3. (PPT) [file pone.0088845.s005.ppt]

## Slide 1
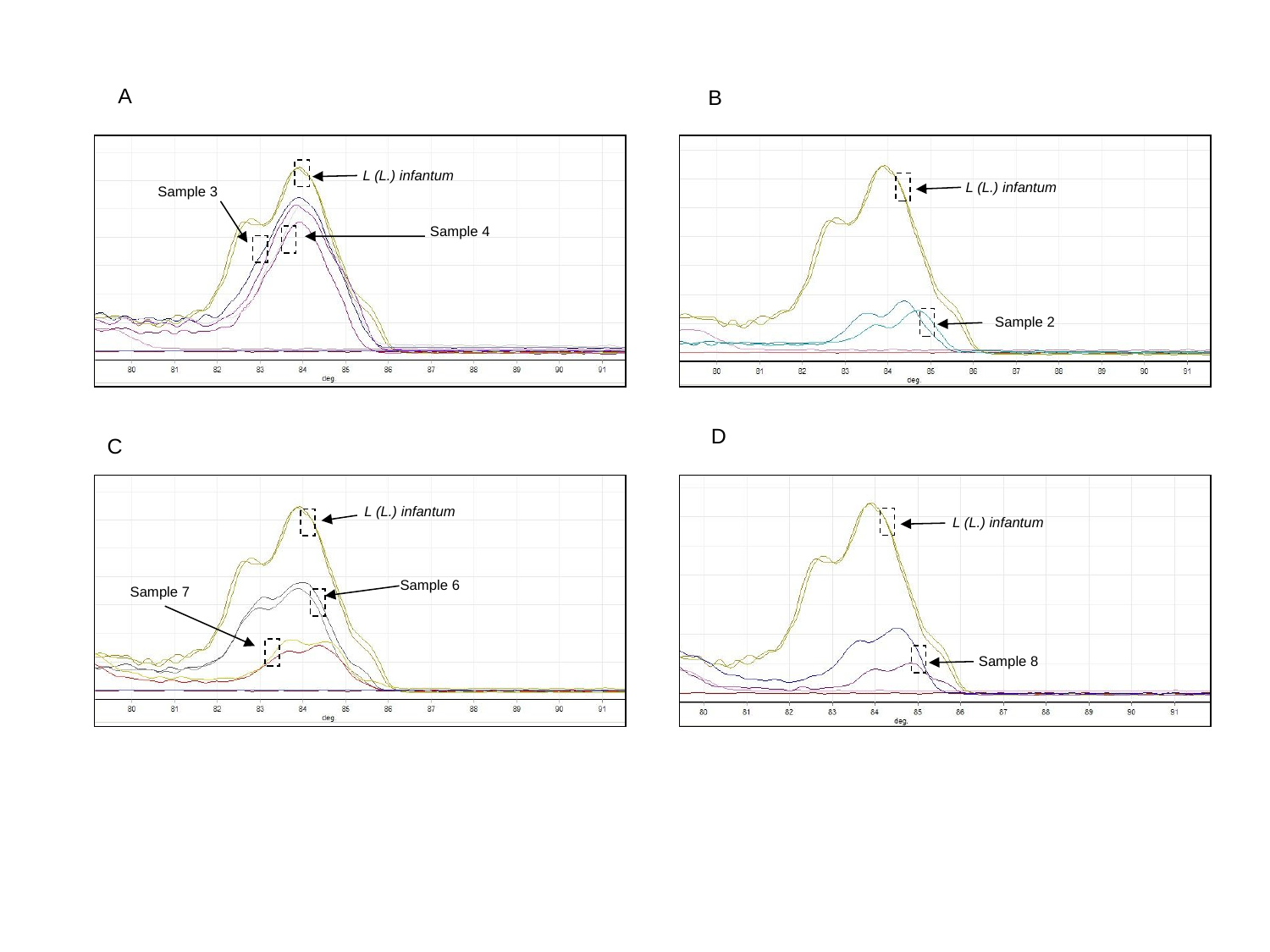

A
B
L (L.) infantum
Sample 2
L (L.) infantum
Sample 3
Sample 4
D
C
L (L.) infantum
Sample 8
L (L.) infantum
Sample 6
Sample 7
